# rGO and rGO/Fullerene Coatings on Blended Textile Fabrics: Characterization of Doctor Blade and Dip Coating Methods

**DOI:** 10.3390/polym18141708

**Published:** 2026-07-11

**Authors:** Dilek Kurt, Umut Kivanc Sahin

**Affiliations:** Faculty of Textile Technologies and Design, Istanbul Technical University, Istanbul 34437, Turkey; kurtd22@itu.edu.tr

**Keywords:** rGO, fullerene, doctor blade coating, dip coating, textile surfaces

## Abstract

Coating method significantly influences how graphene derivatives perform on textile fibers, especially blended substrates where fiber chemistry varies. Graphene oxide (GO) and GO/fullerene composites were deposited on a blended fabric (45% PET, 25% viscose, 8% elastane, 22% recycled PET) using doctor blade and dip coating, followed by chemical reduction to rGO. Three thickness levels were applied for each method: blade gap settings of 50, 100, and 150 µm for doctor blade coating, and immersion times of 30 s, 2 min, and 5 min for dip coating. A more concentrated, higher-viscosity GO dispersion was prepared for doctor blade coating. FTIR spectra showed spectral changes consistent with chemical reduction in GO, including attenuation of hydroxyl-related absorption bands. Electrical resistivity and mechanical properties were determined via conductivity measurements and tensile testing. Doctor blade coatings achieved 0.81 kΩ/sq for rGO/fullerene at the highest thickness, 27 to 70 times lower than dip-coated samples (22.1–56.9 kΩ/sq), depending on coating material and level. The difference reflects uniform blade deposition on hydrophobic polyester fibers, whereas immersion leads to uneven particle distribution. Fullerene addition reduced sheet resistance by approximately fivefold. Mechanical testing showed that coating did not degrade fabric integrity; elongation values remained above 70% across all samples, with most exceeding 80%. These results suggest that, under the present processing conditions, the lower sheet resistance was mainly related to the more continuous coating morphology obtained by doctor blade coating.

## 1. Introduction

Electrically conductive textiles have become an active area of development in wearable technology, with applications ranging from body temperature monitoring and motion sensing to Joule heating [1,2]. Integrating electrical function into fabric structures without compromising flexibility or processability remains a central challenge. Surface coating with conductive materials addresses this directly: it adds electrical functionality to existing textile substrates without requiring fundamental changes to standard manufacturing processes [3].

Among carbon-based materials considered for this application, graphene offers exceptional electrical conductivity and outstanding mechanical properties [4,5]. These electrical and mechanical properties originate from the hexagonal lattice formed by sp^2^ hybridized carbon atoms [6]. Single-layer graphene has been experimentally shown to reach thermal conductivity values exceeding 5000 W/mK at room temperature [7]. Its direct use in textile coatings is, however, limited by poor dispersibility in aqueous media—a practical constraint for most wet-coating processes. Graphene oxide (GO) circumvents this: the oxygen-bearing functional groups on its surface render it water-dispersible and compatible with standard wet-processing equipment [8]. These properties have enabled the widespread use of GO in various functional applications, including wearable textile sensors and supercapacitors [3].

Since GO is electrically insulating, chemical reduction to reduced graphene oxide (rGO) is required to restore the conjugated carbon network and recover conductivity. Ascorbic acid is commonly used for this purpose, owing to its low toxicity and compatibility with textile substrates [9].

The electrical performance of an rGO-coated textile is governed not only by the quantity of deposited material, but also by the continuity of the conductive network formed across the fiber surface. A similar principle has been observed in other graphene-based composite systems; in carboxylated graphene-filled PVA films, the three-dimensional network formed through filler dispersion and interfacial interaction has been shown to govern material performance [10]. Fullerene C60—a spherical carbon allotrope with nanometer-scale dimensions—has been proposed as a structurally complementary additive. Its geometry allows C60 molecules to occupy inter-sheet voids within the rGO network, potentially improving percolation pathways and reducing sheet resistance [11]. C60 has been deposited on textile surfaces in earlier work: Keskin et al. reported increased tensile strength on para-aramid fabrics coated with C60 thin films [12]. The combined effect of rGO and C60 on the electrical properties of coated textiles has, however, not been reported.

Considerable work has been done on rGO coatings applied to cotton and cotton-blend fabrics. Kocanalı and Apaydın Varol obtained surface resistance values as low as 8.18 kΩ on rGO-coated cotton after 90 dip-dry cycles [13]. Özen et al. achieved resistance below 100 kΩ on a woven cotton/polyester blend and demonstrated conductivity retention after repeated washing and mechanical deformation [14]. Gultekin reported simultaneous improvements in electrical conductivity, UV protection, and hydrophobicity on rGO-coated cotton [15,16]. Evseev et al. demonstrated that electrochemically exfoliated graphene coatings on cotton retain conductivity after repeated washing cycles, highlighting the durability potential of graphene-based e-textiles [17]. Using an electrochemical deposition method, Jafari and Botte obtained Joule heating above 120 °C at low applied voltage alongside antibacterial activity [2]. rGO coatings thus appear capable of delivering both electrical conductivity and secondary functional properties within a single processing step.

Most of these studies, however, were conducted on single-component substrates. Blended fabrics have received comparatively little attention in the graphene-coating literature [14], with reports limited mostly to GO-coated wool/synthetic blends [18]. The hydrophobic character of polyester fibers, common in blended constructions, is a relevant factor: GO particles do not adhere to polyester as readily as to cotton, which affects both coating uniformity and the resistance values achievable [19].

The coating method is an equally underexamined variable. Most published work employs a single deposition technique; direct comparisons between methods on the same substrate under identical conditions are rare. Doctor blade and dip coating are both established in textile finishing, but they operate by different mechanisms: blade coating deposits a mechanically controlled film, while dip coating depends on the fabric’s capacity to absorb and retain the dispersion. Comparisons across coating routes such as dip coating, spray coating, airbrushing, and filtration have shown that the deposition method strongly affects the uniformity and conductivity of the resulting graphene layer [20,21,22,23,24]. On a PET-rich blended substrate, where fiber surface energy constrains pickup, this distinction is likely to be consequential [13,14], an issue also reported for spray-coated GO on polyester fabric [21]. The rGO/fullerene combination on blended textiles adds a further unresolved question [11].

The present study addresses these gaps by coating a blended textile fabric (45% PET/25% viscose/8% elastane/22% recycled PET) with GO and GO/fullerene dispersions, followed by chemical reduction to rGO, using both doctor blade and dip coating at three thickness levels. The effects of coating material, method, and thickness on electrical and mechanical properties were evaluated to determine which processing variables exert the strongest influence on functional performance.

Studies on blended fabrics remain limited in this area, despite the substrate used here being a commercial fabric. Furthermore, studies combining both fullerene and GO in a single coating, together with a direct comparison of coating methods, are similarly scarce: to date, none have been reported. This combination of factors makes the present study highly original.

A few quantitative parameters keep the coating method separate from the other variables here. Doctor blade dispersions needed five times more GO than dip coating ones, simply to get a paste thick enough to spread with the blade. Fullerene-to-GO ratio stayed under 1% in both cases—0.33% for doctor blade, 0.5% for dip coating. So, any gap between rGO and rGO/fullerene samples is not coming from one dispersion being loaded with far more fullerene than the other. Three thickness levels were set for each method too, either by blade gap (50, 100, 150 µm) or immersion time (30 s, 2 min, 5 min). Weight gain was recorded for every sample. This lets the amount of material deposited to be checked separately from the method itself, something that earlier comparisons on blended fabrics did not really do.

## 2. Materials and Methods

The substrate was a blended textile fabric containing 45% polyester (PET), 25% viscose, 8% elastane, and 22% recycled PET fiber according to the product label. No pre-treatment was applied before coating.

Graphene oxide (GO) and fullerene (C60) served as the coating materials; both were used as received, without further purification. GO was supplied by Progen Kimya (İstanbul, Turkiye) with a purity of 99%, a surface area of 15.62 m^2^/g, and 2–5 layers. Fullerene (C60) was supplied by Latro (İstanbul, Turkey). Distilled water was used as the solvent in all dispersions.

Two types of coating dispersions were prepared: a GO dispersion and a GO/fullerene composite dispersion.

For dip coating, 1.0 g of GO was dispersed in 200 mL of distilled water. For the GO/fullerene composite dispersion, 1.0 g of GO and 0.005 g of fullerene were dispersed in 200 mL of distilled water. For doctor blade coating, 1.5 g of GO was dispersed in 60 mL of distilled water. For the composite dispersion, 1.5 g of GO and 0.005 g of fullerene were dispersed in 60 mL of distilled water. The higher GO concentration in the doctor blade dispersions was needed to get a paste-like consistency for blade application.

**Figure 1:**
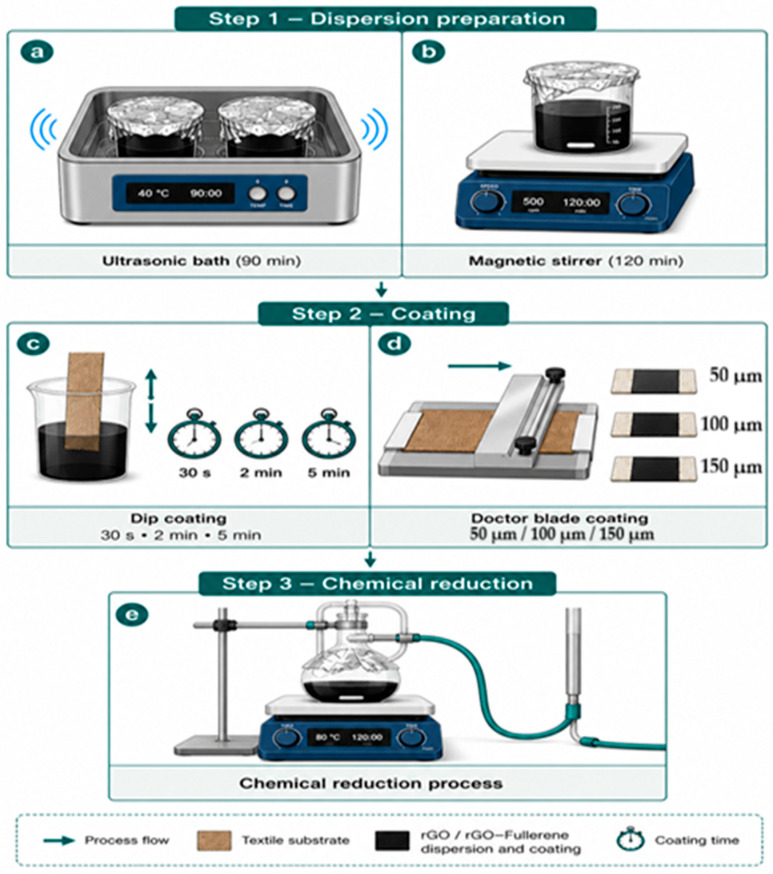
Experimental process: (**a**) ultrasonic bath, (**b**) magnetic stirrer for dispersion preparation, (**c**) dip coating, (**d**) doctor blade coating, and (**e**) chemical reduction in water bath at 95 °C.

All four dispersions were prepared identically: 90 min in an ultrasonic bath, (ISOLAB Laborgeräte GmbH, Eschau, Germany) followed by 120 min on a magnetic stirrer (Daihan Scientific Co., Ltd., Wonju-Si, Republic of Korea). The doctor blade dispersion was visibly thicker than the dip coating formulation—a direct consequence of the higher GO concentration. Neither dispersion showed sedimentation or foaming after preparation, and both were ready to apply without further adjustment. Two coating methods were used: dip coating and doctor blade coating. The overall experimental steps, comprising dispersion preparation, coating, and chemical reduction, are shown schematically in Figure 1. Each method was applied at three levels. After coating, all samples were dried at 60 °C for 10 min in an oven. For dip coating, the fabric samples were immersed in the dispersion and passed through a padding mangle at 3 m/min and 2 bar pressure. Three immersion times were applied: 30 s (level I), 2 min (level II), and 5 min (level III). The fabric showed faint streak-like marks after withdrawal from the dispersion, which became slightly more visible after drying; these marks persisted regardless of the number of mangle passes. For doctor blade coating, the dispersion spread rapidly across the fabric surface with minimal overflow at the edges. A 0.5 mm gap blade was used to spread the dispersion across the fabric surface. The process was repeated to obtain three coating thicknesses: 50 µm (level I), 100 µm (level II), and 150 µm (level III). The gap between the blade and the fabric surface was set using a dial gauge with 0.01 mm precision, and the 50, 100, and 150 µm values reported here correspond to this gap setting. The actual post-drying coating thickness was also measured. Thickness increase ranged from 2.3% to 5.6% for doctor blade samples and from 0.6% to 2.7% for dip-coated samples, a difference consistent with the surface deposition contrast between the two methods.

Twelve coated samples were produced, plus one uncoated control. The sample codes are based on the coating method (DC = dip coating, DB = doctor blade), the coating material (R = rGO, RF = rGO/fullerene), and the coating level (I, II, III). The samples are listed in Table 1.

After coating, we reduced all samples chemically using ascorbic acid (vitamin C) in a water bath at a 1:20 liquor ratio. For 53.89 g of fabric, 1077.8 mL of solution was used. The ascorbic acid concentration was 0.2 mol/L, which required 35.2 g of ascorbic acid per liter. The reduction was carried out at 95 °C for 90 min. All coated samples entered the reduction bath with a brown appearance and exited with a dark anthracite color. This color change was consistent across all twelve samples. After reduction, the fabrics were rinsed in distilled water three times, one minute each, with gentle agitation by immersion, to remove residual ascorbic acid before drying.

Sheet resistance was measured with a Fluke 17B+ digital multimeter, (Fluke Corporation, Everett, WA, USA) probes set 1 cm apart. For each sample, three measurements were taken in the warp direction and three in the weft direction, each from a different location on the fabric. The reported values are the average of these readings (mean ± SD, *n* = 6). Prior to testing, all samples were conditioned at 20 °C and 65% relative humidity for 24 h.

Tensile tests were carried out on a Zwick Roell Z005 testing machine (ZwickRoell GmbH & Co. KG, Ulm, Germany) fitted with a 100 N load cell. Six specimens were prepared per sample: three in the warp direction, three in the weft. Each specimen measured 1 cm by 5 cm. Across the twelve coated groups, this gave 72 specimens in total. Specimens were loaded and tested one at a time, with results recorded as each test finished. Conditioning followed the same protocol as the electrical measurements: 20 °C, 65% relative humidity, 24 h.

## 3. Results

### 3.1. Electrical Properties

**Figure 2:**
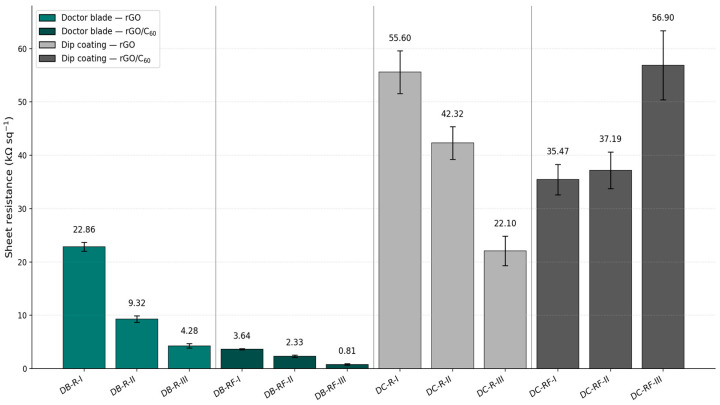
Sheet resistance values of coated fabric samples. Doctor blade groups (DB) show consistently lower resistance than dip coating groups (DC). The lowest value (0.81 kΩ/sq) was obtained for DB-RF-III. Error bars represent ±SD (*n* = 6: three measurements in the warp direction and three in the weft direction per sample).

Sheet resistance values for all twelve samples are presented in Figure 2. Doctor blade samples showed lower resistance than dip-coated samples across all groups and coating levels. The lowest value was 0.81 kΩ/sq, recorded for DB-RF-III. The highest was 56.9 kΩ/sq, recorded for DC-RF-III. Within the doctor blade groups, resistance decreased with increasing coating level. No clear trend was observed in dip-coated samples.

The addition of C60 to the rGO dispersion reduced sheet resistance in doctor blade samples at all coating levels. At level I, DB-RF-I recorded 3.64 kΩ/sq against 22.86 kΩ/sq for DB-R-I. At level III, DB-RF-III reached 0.81 kΩ/sq against 4.28 kΩ/sq for DB-R-III. No equivalent reduction was observed in dip-coated samples, where values remained high regardless of fullerene addition.

To clarify the contribution of the coating method independent of the dispersion concentration used, dry weight gain (coating add-on) was determined for each sample by weighing the fabric before and after coating. Table 2 presents these values alongside the corresponding sheet resistance. The average weight gain was 1.63% for doctor blade samples and 0.88% for dip-coated samples, a 1.85-fold difference, considerably smaller than the five-fold difference in dispersion concentration. The sheet resistance difference between the lowest- and highest- performing samples (DB-RF-III and DC-RF-III) was 70-fold, while the corresponding weight gain difference was only 1.9-fold (1.7% versus 0.9%). The magnitude of the resistance difference is therefore not proportional to the amount of material deposited, indicating that coating method governs sheet resistance primarily through the continuity and morphology of the deposited layer rather than through deposited mass alone. The fullerene-to-GO ratio also differed between the two dispersions (0.33% for doctor blade, 0.5% for dip coating), so the dip-coated dispersions were relatively richer in fullerene relative to GO, not poorer. Despite this, doctor blade samples still achieved the lower sheet resistance, which argues against the composition ratio being the determining factor and further supports coating morphology as the primary driver.

### 3.2. Mechanical Properties

**Figure 3:**
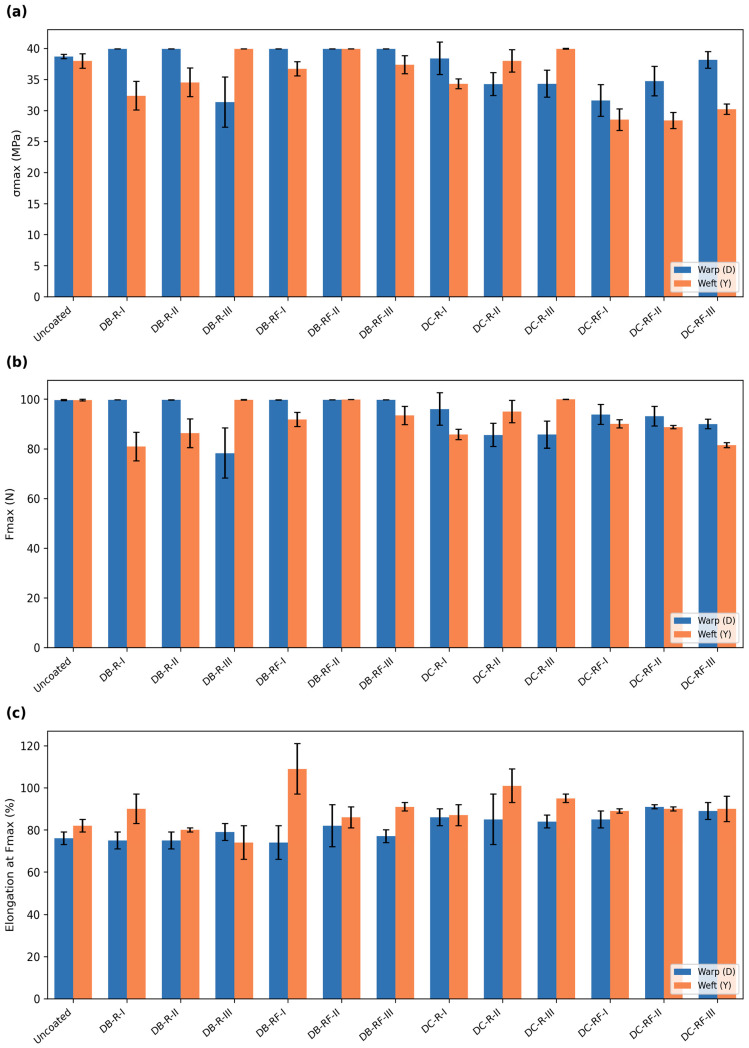
Tensile properties of coated fabric samples in warp (D) and weft (Y) directions: (**a**) maximum stress σmax, (**b**) maximum force Fmax, and (**c**) elongation at Fmax. Error bars represent ±SD (*n* = 3).

Tensile test results of all samples are presented in Table 3 and Figure 3. Most samples reached the 100 N force limit, and these results show that the coating and reduction processes did not damage the fabric. An uncoated fabric sample was also tested for comparison. The maximum force of the uncoated sample (99.60 ± 0.19 N warp, 99.60 ± 0.30 N weft) was also close to the 100 N limit, which shows that this limit comes from the fabric structure itself, not from the coating process. The actual maximum force values, for both the uncoated and coated samples, are likely higher than the values reported here; a tensile tester with a higher force capacity would be needed to determine these values. The maximum stress (38.70 ± 0.36 MPa warp, 37.97 ± 1.16 MPa weft) and elongation (76 ± 3% warp, 82 ± 3% weft) of the uncoated sample were similar to the most of the coated groups, which supports the conclusion that the coating process did not weaken the fabric.

In most groups, the warp direction showed higher stress values, and the weft direction showed higher elongation values. However, some groups did not follow this trend. In the DB-R-III, DC-R-II, and DC-R-III groups, the weft direction showed a higher stress value than the warp direction. In the DB-RF-II group, no difference was observed between the two directions, and both reached the 39.90 MPa limit. In the DB-R-III and DC-RF-I groups, the warp direction showed higher elongation than the weft direction.

The DB-RF groups remained close to the stress limit even after the addition of fullerene. The DC-RF groups did not show this behavior: the DC-RF-I, DC-RF-II, and DC-RF-III groups all decreased to the 28–35 MPa range in both directions, below most of the DC-R values. This shows that the negative effect of fullerene on tensile strength only appears clearly in the dip coating method, not in the doctor blade method. This result is consistent with the structural and penetration differences discussed elsewhere in this study.

### 3.3. FTIR Analysis

FTIR spectra were recorded using a PerkinElmer Spectrum Two FT-IR spectrometer to evaluate the chemical structure of the uncoated and coated fabric samples.

**Figure 4:**
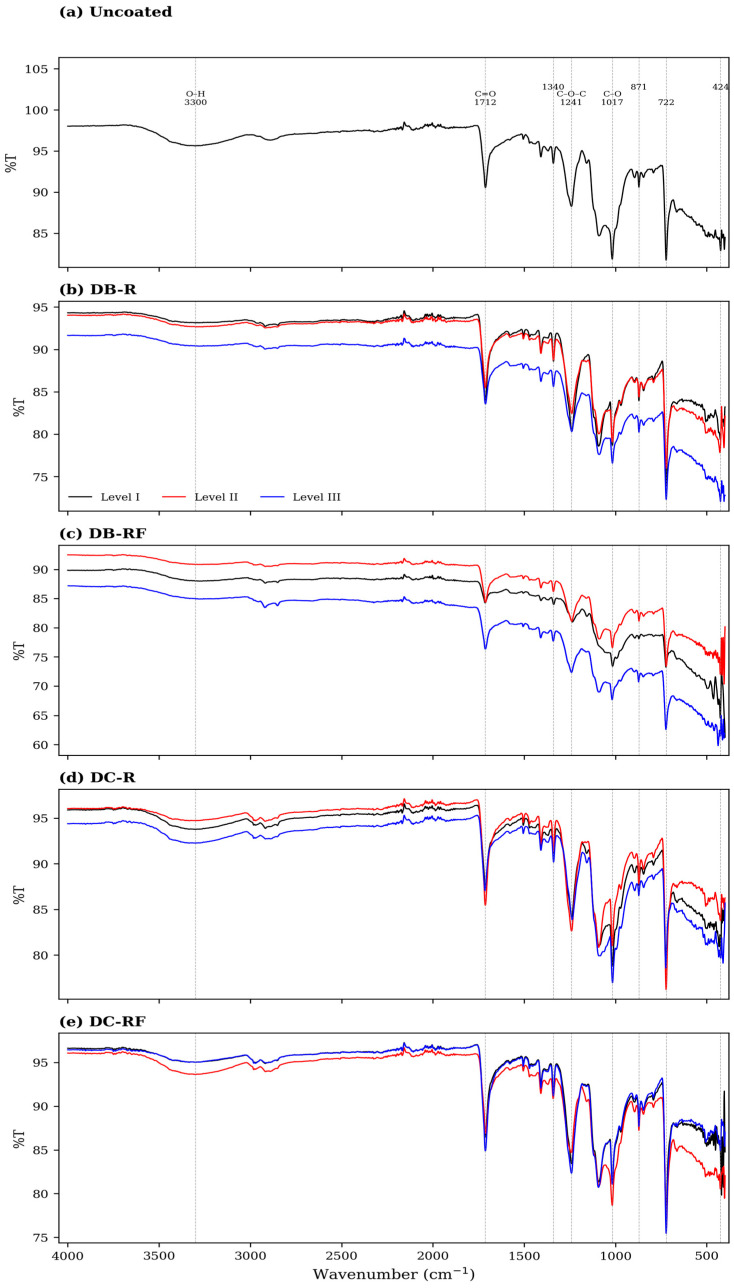
FTIR spectra of coated fabric samples at three coating concentrations (I, II, III) for each group: (**a**) Uncoated Fabric; (**b**) DB-R; (**c**) DB-RF; (**d**) DC-R; (**e**) DC-RF. Black lines: Level I; red lines: Level II; blue lines: Level III.

FTIR spectra of the uncoated fabric and all twelve coated samples are presented in Figure 4, grouped by coating formulation. The uncoated fabric displays the typical absorption pattern of a polyester-containing substrate. All coated samples retain these bands, confirming that neither the coating nor the reduction process chemically altered the fabric. In the DB-R group (Figure 4b), transmittance decreases progressively with increasing coating concentration, most noticeably in the Level III sample.

A stronger reduction is observed in the DB-RF group (Figure 4c), particularly below 1500 cm^−1^. Denser and more absorbing, the DB-RF coating layer reflects the contribution of C60 to the composite structure. These findings are consistent with the SEM observations and the lower sheet resistance values recorded for the doctor blade groups. The dip-coated samples present a contrasting picture. In both DC-R (Figure 4d) and DC-RF (Figure 4e), increasing the coating concentration makes little difference, as the spectra remain close to the uncoated control at all three levels. Little of the coating material, it appears, reached the fiber surface. The high sheet resistance values measured for these groups point to the same conclusion. None of the coated samples show signs of unreduced GO. Around 3300 cm^−1^ and 1060 cm^−1^, no new peaks appear, which is consistent with the ascorbic acid treatment having reduced the GO [9].

### 3.4. SEM Analysis

**Figure 5:**
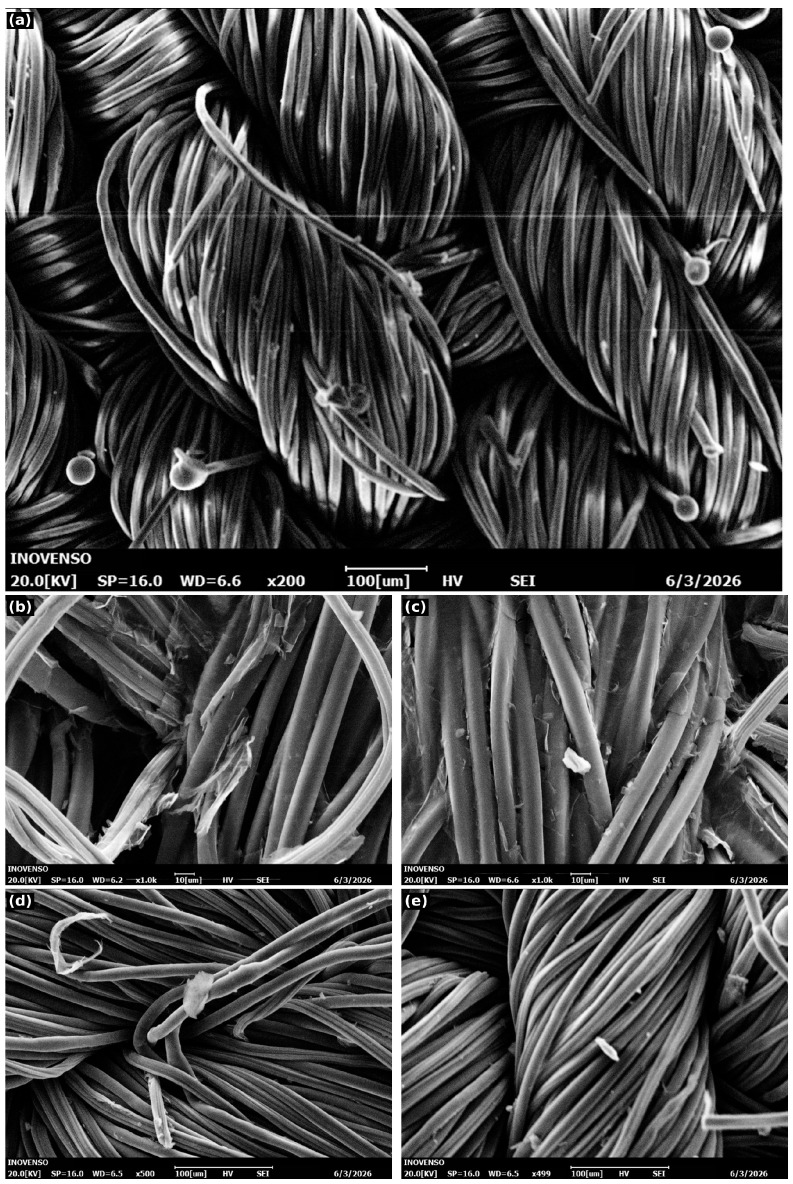
SEM micrographs of (**a**) uncoated control fabric; (**b**) DB-R-III; (**c**) DB-RF-III; (**d**) DC-R-III; (**e**) DC-RF-III.

The surface morphology of the uncoated fabric and four selected samples (DB-R-III, DB-RF-III, DC-R-III, and DC-RF-III) was examined by SEM. Representative micrographs are shown in Figure 5.

The uncoated fabric (Figure 5a) has clean, smooth fiber surfaces. The small, rounded features visible at some fiber ends are filament cut ends, not deposits.

In DB-R-III (Figure 5b), thin flat films cover parts of the fiber surface. Sheet-like, not spherical, these deposits are characteristic of rGO. Coverage is not complete across all fibers, but the presence of continuous film regions shows that the doctor blade method was able to transfer rGO onto the substrate.

DB-RF-III (Figure 5c) shows a denser deposit. Alongside the flat rGO film, a small irregular, flake-like particle is visible on the fiber surface. Its size and angular shape are inconsistent with an individual C60 molecule, which is roughly 0.7 nm across, so its origin cannot be confirmed as fullerene-related from SEM morphology alone; it is more consistent with surface contamination. The sheet resistance trend itself, dropping from 4.28 kΩ/sq in DB-R-III to 0.81 kΩ/sq in DB-RF-III, is the stronger evidence for a fullerene contribution.

DC-R-III (Figure 5d) tells a different story. The fiber surfaces are largely bare. Dip coating, it seems, could not hold the rGO dispersion on the hydrophobic PET-rich substrate, unlike the doctor blade method where mechanical spreading forced the material onto the surface.

In DC-RF-III (Figure 5e), the picture is much the same. A small elongated, flake-like particle sits on an otherwise uncovered fiber, rather than within a continuous film.

A dispersion this poor cannot form a connected fullerene network, and the sheet resistance of 56.9 kΩ/sq reflects that.

## 4. Discussion

Comparing DB-RF-III and DC-RF-III, the difference in weight gain is only 1.9-fold (1.7% versus 0.9%), while the difference in sheet resistance was measured at 70-fold (0.81 kΩ/sq versus 56.9 kΩ/sq). Were the resistance difference due only to the amount of deposited material, this difference would also be expected to be of similar size, around 1.9-fold. But the observed difference is 70-fold. A difference in this size cannot be explained by a 1.9-fold weight difference. This shows the main factor determining resistance is not the amount of deposited material, but how the material is distributed across the surface. Within the dip coating group, a notable result is also present: the DC-RF-III sample has both the highest weight gain (0.9%) and the highest resistance (56.9 kΩ/sq) among the rGO/fullerene dip-coated samples. A decrease in resistance would be expected as deposited material increases, but the opposite was observed here. This result also supports the assessment that distribution, not amount, is the determining factor for resistance. The effect of filler dispersion and local aggregation on final performance has been similarly reported in other polymer–graphene composite systems; in PBS nanocomposites reinforced with ZnO nanoplatelet-coated graphene, filler dispersion and network structure have been shown to govern mechanical and barrier properties [25], and rGO filler content has likewise been shown to govern crystal structure and degradation behavior in PCL composite films [26]. The repeated sheet resistance measurements support this picture of morphological continuity. Doctor blade samples showed low variability across the three measured locations (SD 0.11–0.84 kΩ/sq), consistent with a uniform film covering the fiber surface. Dip-coated samples showed substantially higher variability (SD 2.77–6.48 kΩ/sq), reflecting the patchy, location-dependent coverage expected when the fabric’s hydrophobic PET-rich surface limits dispersion uptake. This difference in measurement spread reinforces the conclusion that coating uniformity, not deposited quantity, is the primary driver of the resistance gap between the two methods.

The resistance values of the two samples made by different methods are close to each other and they have a comparable amount of deposited material. This points to coating uniformity, not the method itself, as the main factor behind the outcome. When the amount of material taken up by the fabric is the same, results can stay close even with different methods, as long as a uniform coating is achieved. The method, then, does not act on the result directly. It acts through the morphology of the coating it produces.

The fivefold resistance reduction between DB-R-III (4.28 kΩ/sq) and DB-RF-III (0.81 kΩ/sq) warrants attention. Fullerene C60 is electrically conductive and nanometer-scale in size; at the concentrations used here, its molecules are geometrically capable of occupying voids between adjacent rGO sheets within the coating. A denser percolation network—fewer and shorter breaks in the conductive path—would produce exactly the resistance drop observed. This is consistent with reports that C60 can raise the conductivity of an existing filler network once a percolation threshold is reached, both in fullerene-only polymer composites [27] and in C60-filled engineering polymers such as poly (phenylene sulfide) [28]. This effect was pronounced in doctor blade samples but not clearly visible in dip-coated ones, where the underlying coating irregularity likely dominates. The fullerene contribution becomes measurable only when the base coating is already sufficiently uniform.

The strongest hydroxyl band decrease was observed in the DB-RF-I and DB-R-III samples, among all twelve coated groups. FTIR also supports the conversion seen in the doctor blade group. The lowest sheet resistance values also belong to the doctor blade group. Both findings show a more complete and more uniform coating in this group. The DC group shows a more limited change in the FTIR results. This matches the lower amount of material reaching the fiber surface during dip coating. The FTIR results overall support the reduction across the coated samples. They should be read as consistent with reduction. They are not definitive proof of complete conversion from GO to rGO. The persistence of the carbonyl band at 1712 cm^−1^ reflects the residual oxygen functionality typical of ascorbic acid-reduced rGO under mild processing conditions [9].

Mechanically, the coating left the fabric intact. All samples reached the 100 N test limit without fracture, and elongation values remained above 70% across all groups. The anisotropic response—higher stiffness in the warp direction, greater elongation in the weft—was preserved after coating and reduction, indicating that neither the dispersion nor the ascorbic acid bath altered the yarn structure or the weave geometry. The elastane content of the fabric likely contributed to this resilience: the fiber’s elasticity absorbs stress concentrations that might otherwise arise at coating-fiber interfaces.

The electrical performance of DB-RF-III (0.81 kΩ/sq) compares favorably with published values for rGO-coated cotton. Kocanalı and Apaydın Varol reported 8.18 kΩ on cotton after 90 dip-dry cycles [13]; values between 36.94 and 348.34 kΩ/sq were reported for cotton after two to five padding passes [1]. The blended substrate used here is inherently less favorable for graphene adhesion than cotton, which makes the DB-RF-III result notable: it was obtained on a single coating pass, without surface pre-treatment, on a fabric with 45% hydrophobic PET content.

## 5. Conclusions

rGO and rGO/fullerene coatings were deposited on a blended polyester–viscose–elastane fabric using doctor blade and dip coating at three thickness levels, yielding twelve experimental groups. Electrical, mechanical, and surface properties were characterized to determine which processing variables exert the strongest influence on functional performance.

Weight gain measurements showed that the five-fold higher rGO concentration used for doctor blade coating resulted in only a 1.85-fold higher deposited mass on average (1.63% versus 0.88%). This difference is too small to account for the much larger resistance gap between the two methods, pointing to surface distribution of the material as the more decisive factor. Between DB-RF-III and DC-RF-III, the weight gain difference was only 1.9-fold, while the sheet resistance difference reached 70-fold, a gap far too large to be explained by deposited mass alone. Samples with matched weight gains further support this conclusion: DB-R-I and DC-R-III, despite being produced by different methods, showed comparable weight gain and similarly close resistance values. Coating method, then, governs sheet resistance mainly through the continuity and morphology of the deposited layer rather than through the amount of material deposited.

Coating method was the dominant variable. Sheet resistance differences between doctor blade and dip coating groups ranged from 2.4-fold (DC-R-I vs. DB-R-I) to 70-fold (DC-RF-III vs. DB-RF-III), depending on coating material and level. The most consistent advantage of blade coating emerged in rGO/fullerene groups, where doctor blade samples outperformed dip-coated equivalents by approximately 10-fold at level I and 70-fold at level III. On a substrate with 45% PET content, dip coating cannot reliably deposit GO onto hydrophobic fibers; blade coating circumvents this by mechanically forcing the dispersion across the surface.

Fullerene addition reduced sheet resistance fivefold in doctor blade groups—from 4.28 kΩ/sq (DB-R-III) to 0.81 kΩ/sq (DB-RF-III). No equivalent gain was observed in dip-coated samples, where coating irregularity dominates and the fullerene contribution is masked. C60 improves percolation only when the base coating is already sufficiently uniform. FTIR spectra were consistent with the GO-to-rGO conversion across all coated samples: attenuation of the hydroxyl band and emergence of characteristic carbon-network peaks are consistent with successful chemical reduction. The persistence of the carbonyl band at 1712 cm^−1^ reflects the residual oxygen functionality typical of ascorbic acid-reduced rGO under mild processing conditions [9].

The fabric survived both coating and reduction intact. All groups reached the 100 N tensile limit without fracture, elongation exceeded 70% across all groups, and the anisotropic warp–weft response was preserved.

These findings show that coating method governs conductivity mainly through the morphological continuity of the deposited layer, not through deposited mass. The same principle could hold for other graphene derivatives and other textile substrates. The 0.81 kΩ/sq value obtained for DB-RF-III came from a single doctor blade pass, with no surface pre-treatment, on a substrate containing 45% hydrophobic PET. The doctor blade method may therefore offer a practical advantage for similarly challenging blended substrates coated with graphene-based conductive materials.

Future work could test other graphene derivative-substrate combinations. The conductivity reached here points toward a few uses. Wearable strain sensors need a stable, low-resistance path that holds up under repeated bending—the kind of uniform coating discussed throughout this paper. Joule heating is another option; DB-RF-III’s sheet resistance sits in the range other studies report for low-voltage heating in rGO textiles. EMI shielding for protective or technical clothing is a third, since it also needs a continuous conductive layer across the fiber surface. None of this works at scale yet, though. Doctor blade here was a single pass on small samples—moving to roll-to-roll coating would be the next step before any of these applications become realistic. Washing durability and repeated mechanical cycling would need testing first as well.

## Figures and Tables

**Table 1:** Sample codes and coating parameters.

Sample Code	Coating Material	Coating Method	Level
Uncoated	—	—	—
DC-R-I	rGO	Dip coating	30 s
DC-R-II	rGO	Dip coating	2 min
DC-R-III	rGO	Dip coating	5 min
DC-RF-I	rGO/fullerene	Dip coating	30 s
DC-RF-II	rGO/fullerene	Dip coating	2 min
DC-RF-III	rGO/fullerene	Dip coating	5 min
DB-R-I	rGO	Doctor blade	50 µm
DB-R-II	rGO	Doctor blade	100 µm
DB-R-III	rGO	Doctor blade	150 µm
DB-RF-I	rGO/fullerene	Doctor blade	50 µm
DB-RF-II	rGO/fullerene	Doctor blade	100 µm
DB-RF-III	rGO/fullerene	Doctor blade	150 µm

Coating thickness level refers to the blade gap setting for doctor blade coating and to the immersion time for dip coating.

**Table 2:** Dry weight gain (add-on) and corresponding sheet resistance of coated fabric samples.

Sample	Pre-Coating Weight (g)	Post-Coating Weight (g)	Weight Gain (%)	Sheet Resistance (kΩ/sq)	SD (kΩ/sq, *n* = 6)
DB-R-I	5.76	5.83	1.2	22.86	0.84
DB-R-II	5.76	5.85	1.6	9.32	0.60
DB-R-III	5.76	5.90	2.4	4.28	0.42
DB-RF-I	5.81	5.90	1.5	3.64	0.11
DB-RF-II	5.72	5.80	1.4	2.33	0.22
DB-RF-III	5.81	5.91	1.7	0.81	0.12
DC-R-I	5.82	5.87	0.9	55.60	4.01
DC-R-II	5.76	5.82	1.0	42.32	3.06
DC-R-III	5.80	5.87	1.2	22.10	2.77
DC-RF-I	5.96	6.01	0.8	35.47	2.85
DC-RF-II	5.82	5.85	0.5	37.19	3.45
DC-RF-III	5.74	5.79	0.9	56.90	6.48

**Table 3:** Tensile properties of coated fabric samples in warp and weft directions (mean ± SD, *n* = 3).

Sample	σmax (MPa)		Fmax (N)		Elong. at Fmax (%)	
	Warp	Weft	Warp	Weft	Warp	Weft
Uncoated	38.70 ± 0.36	37.97 ± 1.16	99.60 ± 0.19	99.60 ± 0.30	76 ± 3	82 ± 3
DB-R-I	39.90 ± 0.00	32.37 ± 2.32	99.71 ± 0.03	80.90 ± 5.74	75 ± 4	90 ± 7
DB-R-II	39.90 ± 0.00	34.53 ± 2.31	99.69 ± 0.04	86.25 ± 5.77	75 ± 4	80 ± 1
DB-R-III	31.33 ± 4.05	39.90 ± 0.00	78.31 ± 10.03	99.73 ± 0.07	79 ± 4	74 ± 8
DB-RF-I	39.90 ± 0.00	36.70 ± 1.14	99.67 ± 0.03	91.81 ± 2.90	74 ± 8	109 ± 12
DB-RF-II	39.90 ± 0.00	39.90 ± 0.00	99.72 ± 0.04	99.80 ± 0.01	82 ± 10	86 ± 5
DB-RF-III	39.90 ± 0.00	37.37 ± 1.45	99.72 ± 0.01	93.43 ± 3.69	77 ± 3	91 ± 2
DC-R-I	38.40 ± 2.60	34.30 ± 0.80	95.99 ± 6.54	85.72 ± 2.07	86 ± 4	87 ± 5
DC-R-II	34.23 ± 1.85	37.97 ± 1.81	85.57 ± 4.64	94.97 ± 4.54	85 ± 12	101 ± 8
DC-R-III	34.30 ± 2.17	39.93 ± 0.06	85.72 ± 5.47	99.89 ± 0.06	84 ± 3	95 ± 2
DC-RF-I	31.60 ± 2.55	28.51 ± 1.73	93.81 ± 4.00	90.05 ± 1.64	85 ± 4	89 ± 1
DC-RF-II	34.73 ± 2.38	28.38 ± 1.28	93.12 ± 3.99	88.70 ± 0.63	91 ± 1	90 ± 1
DC-RF-III	38.14 ± 1.35	30.20 ± 0.85	89.94 ± 1.93	81.47 ± 0.98	89 ± 4	90 ± 6

## Data Availability

The original contributions presented in this study are included in the article. Further inquiries can be directed to the corresponding author.

## Abbreviations

The following abbreviations are used in this manuscript:

GO	Graphene oxide
rGO	Reduced graphene oxide
C60	Fullerene C60
PET	Polyester
SEM	Scanning electron microscopy
FTIR	Fourier-transform infrared spectroscopy
DB	Doctor blade coating
DC	Dip coating
